# Ethyl 2-[4-(4-meth­oxy­benz­yl)-3-methyl-6-oxopyridazin-1-yl]acetate

**DOI:** 10.1107/S241431462200582X

**Published:** 2022-06-07

**Authors:** Younes Zaoui, Hamza Assila, Joel T Mague, Abdulsalam Alsubari, Jamal Taoufik, Youssef Ramli, Mhammed Ansar

**Affiliations:** a Laboratory of Medicinal Chemistry, Drug Sciences Research Center, Faculty of Medicine and Pharmacy, Mohammed V University in Rabat, Rabat, Morocco; b Department of Chemistry, Tulane University, New Orleans, LA 70118, USA; c Laboratory of Medicinal Chemistry, Faculty of Clinical Pharmacy, 21 September University, Yemen; Sunway University, Malaysia

**Keywords:** crystal structure, di­hydro­pyridazine, hydrogen bond, π-stacking

## Abstract

In the title mol­ecule, C_17_H_20_N_2_O_4_, the inner part of the ester substituent is nearly perpendicular to the di­hydro­pyridazine ring, forming a dihedral angle of 83.21 (7)°. In the crystal, inversion dimers are formed by pairwise C—H⋯O inter­actions with the dimers connected into chains extending along the *b*-axis direction by C—H⋯π(ring) inter­actions. The chains are connected by π-stacking inter­actions to give corrugated layers parallel to the *ab* plane. The terminal ethyl group is disordered over two two sets of sites with the major component having a site occupancy factor of 0.715 (10)

## Abstract 2

The inner part of the ester substituent is nearly perpendicular to the di­hydro­pyridazine ring while the *ipso* carbon of the 4-meth­oxy­phenyl group is close to the above plane. In the crystal, corrugated layers parallel to the *ab* plane are formed by a combination of C—H⋯O, C—H⋯π(ring) and π-stacking inter­actions.

## Graphical abstract

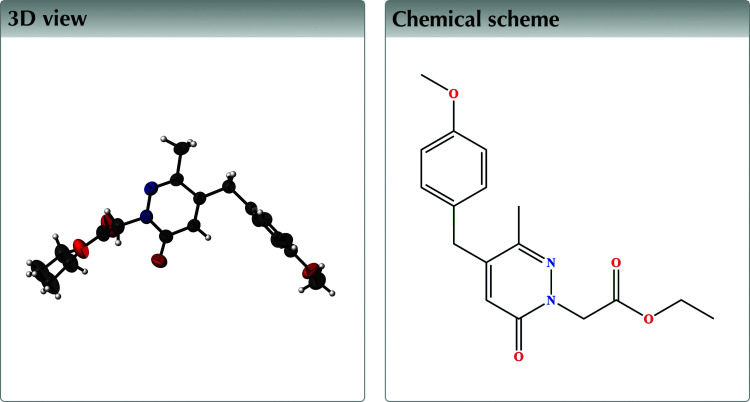

## Structure description

**Figure 1:**
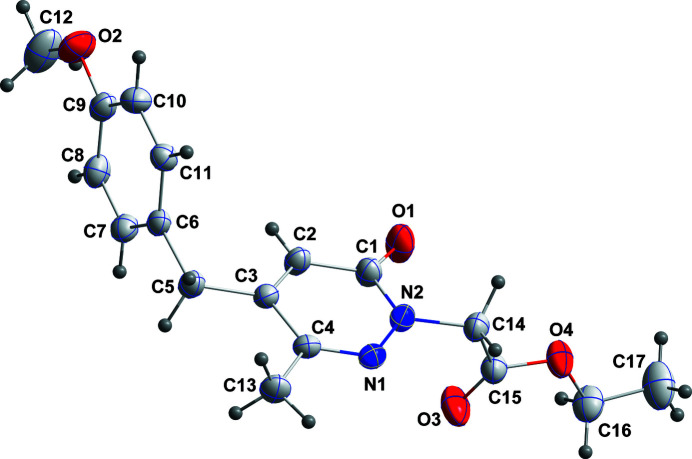
The title mol­ecule with labelling scheme and 30% probability ellipsoids. Only the major component of the disordered ethyl group is shown.

Pyridazinone derivatives, with a carbonyl group at position 3, possess a number of biological activities including anti-oxidant (Khokra *et al.*, 2016 ▸), anti-bacterial and anti-fungal (Abiha *et al.* 2018 ▸), anti-cancer (Kamble *et al.* 2017 ▸), analgesic and anti-inflammatory (Ibrahim *et al.* 2017 ▸), anti-depressant (Boukharsa *et al.* 2016 ▸) and anti-ulcer activities (Yamada *et al.*, 1981 ▸). In addition, a number of pyridazinone derivatives have been reported to have potential as agrochemicals, for example as insecticides (Nauen & Bretschneider, 2002 ▸). As part of our ongoing studies of these systems, we report herein the synthesis and the mol­ecular and crystal structure of the title compound (Fig. 1 ▸).

The dihedral angle between the N1/N2/C1–C4 and C6–C11 planes is 89.74 (3)° while that between the N1/N2/C1–C4 plane and that defined by N2/C14/C15/O3 is 83.21 (7)°. This latter angle indicates that the inner end of the substituent on N2 is nearly perpendicular to the tetra­hydro­pyridazine ring. The C2—C3—C5—C6 torsion angle of −9.4 (2)° indicates that the centroid of the 4-meth­oxy­phenyl ring is only slightly below the plane of the pyridazine ring. This conformation appears to be the result of the inter­molecular π-stacking inter­action (see below).

**Figure 2:**
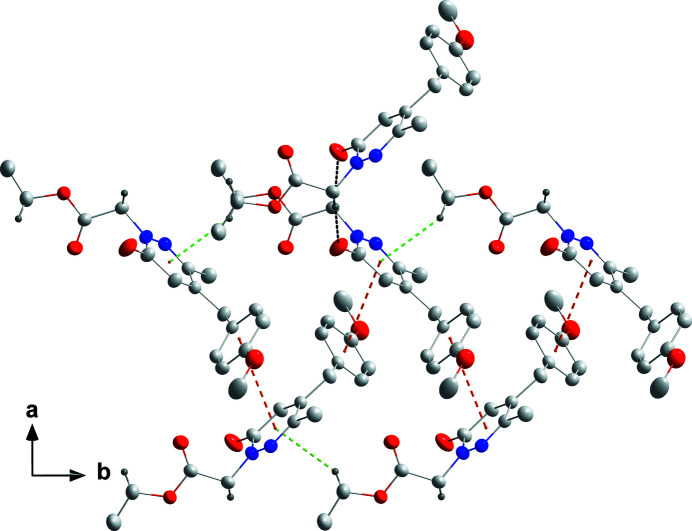
Detail of the inter­molecular inter­actions viewed along the *c*-axis direction. C—H⋯O hydrogen bonds are shown by black dashed lines while π-stacking and C—H⋯π(ring) inter­actions are shown, respectively, by orange and green dashed lines.

In the crystal, inversion dimers are formed by pairwiseC14—H14*B*⋯O1 inter­actions (Table 1 ▸) with the dimers connected into chains extending along the *b*-axis direction by C16—H16*B*⋯*Cg*1 inter­actions (Table 1 ▸ and Fig. 2 ▸). The chains are connected to one another by π-stacking inter­actions between the N1/N2/C1–C4 and C6^i^–C11^i^ rings [symmetry code: (i) −*x* + 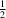 , *y* + 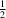 , −*z* + 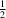 ] with a centroid–centroid distance of 3.8870 (8) Å and a dihedral angle of 7.29 (6)° to give corrugated layers parallel to the *ab* plane (Figs. 2 ▸ and 3 ▸).

## Synthesis and crystallization

A mixture of 3-(4-meth­oxy­benzyl­idene)-4-oxo­penta­noic acid (0.05 mol) and hydrazine hydrate (0.1 mol) in ethanol (100 ml) was refluxed for 2 h. The precipitate that formed was filtered off and recrystallized from acetone solution to obtain the 5-(4-meth­oxy­benz­yl)-6-methyl­pyridazin-3(2*H*)-one pre­cursor. To this pyridazine derivative (0.05 mol) was added potassium carbonate (0.1 mmol), tetra­butyl­ammonium bromide (0.01 mmol) and 2-ethyl bromo­acetate (0.1 mol) in di­methyl­formamide (20 ml). The mixture was stirred for 24 h at room temperature. At the end of the reaction, the solution was filtered and the solvent evaporated under reduced pressure. The residue was washed with water and methyl­ene chloride. The solvent was removed and colourless blocks of the title compound were obtained by recrystallization of the product from its acetone solution.

Yield 79%; m.p. 406–408 K. IR (cm^−1^): 1743 (C=O, CO_2_Et), 1660 (C=ON), 1599 (C=C), 1205 (C—N), 1011 and 1145 (C—O, CO_2_Et *sym* and *asym*). ^1^H NMR (p.p.m.): 1.23 (*t*, 3H, *J* = 7.1, CH_2_—CH_3_); 2.22 (*s*, 3H, CH_3_-pyridazinone); 2.33 (*s*, 3H, OCH_3_-phen­yl); 3.85 (*s*, 2H, phenyl-CH_2_-pyridazinone); 4.17 (*q*, 2H, *J* = 7.1, O—CH_2_—CH_3_); 4.87 (*s*, 2H, –N—CH_2_—CO); 6.48 (*s*, 1H, pyridazinone); 6.93–6.96 (*d*, 2H, *J =* 9, phen­yl); 7.25–7.27 (*d*, 2H, *J =* 9, phen­yl). ^13^C NMR (p.p.m.): 14.11 (CH_3_); 21.03 (CH_3_, pyridazinone); 25.21 (OCH_3_, phen­yl); 37.67 (CH_2_); 51.34 (CH_2_); 60.95 (CH_2_); 127.13–127.44 (CH aromatic); 129.13–130.35 (CH aromatic); 132.12 (C—Cα aromatic); 136.51 (CH_2_—C=, aromatic); 138.49 (CH, pyridazinone); 144.97 (CH_2_—C=CH, pyridazinone); 147.17 (C=N); 161.19 (C=O, pyridazinone); 169.52 (C=O, CO_2_Et).

## Refinement

Crystal data, data collection and structure refinement details are summarized in Table 2 ▸. The C16/C17 ethyl group is disordered and was refined as two components restrained to have comparable geometries. The refined occupancies were 0.715 (10) and 0.285 (10).

## Supplementary Material

Crystal structure: contains datablock(s) global, I. DOI: 10.1107/S241431462200582X/tk4077sup1.cif

Structure factors: contains datablock(s) I. DOI: 10.1107/S241431462200582X/tk4077Isup2.hkl

Supporting information file. DOI: 10.1107/S241431462200582X/tk4077Isup3.cml

CCDC reference: 2175897

Additional supporting information:  crystallographic information; 3D view; checkCIF report

## Figures and Tables

**Figure 3:**
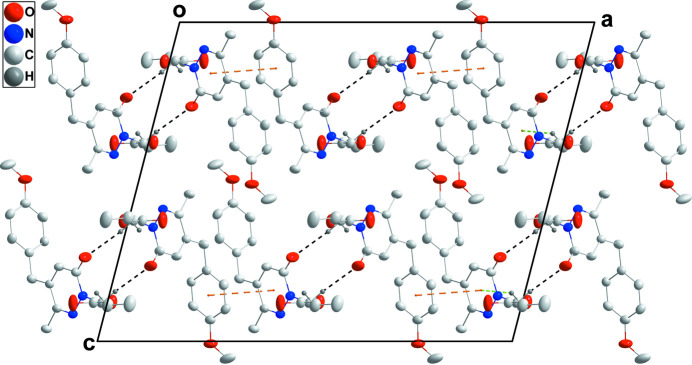
Packing viewed along the *b*-axis direction with the highlighted inter­molecular inter­actions shown as in Fig. 2 ▸.

**Table 1:** Hydrogen-bond geometry (Å, °) *Cg*1 is the centroid of the C1–C4/N1/N2 ring.

*D*—H⋯*A*	*D*—H	H⋯*A*	*D*⋯*A*	*D*—H⋯*A*
C14—H14*B*⋯O1^i^	0.97	2.44	3.4041 (19)	175
C16—H16*B*⋯*Cg*1^ii^	0.97	2.86	3.586 (3)	132

Symmetry codes: (i) 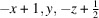 ; (ii) 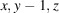 .

**Table 2:** Experimental details

Crystal data	
Chemical formula	C_17_H_20_N_2_O_4_
*M*_r_	316.35
Crystal system, space group	Monoclinic, *C*2/*c*
Temperature (K)	298
*a*, *b*, *c* (Å)	23.0488 (9), 8.1149 (3), 18.3223 (7)
β (°)	104.454 (1)
*V* (Å^3^)	3318.5 (2)
*Z*	8
Radiation type	Mo *K*α
μ (mm^−1^)	0.09
Crystal size (mm)	0.30 × 0.27 × 0.26

Data collection	
Diffractometer	Bruker SMART APEX CCD
Absorption correction	Multi-scan (*SADABS*; Krause *et al.*, 2015 ▸)
*T*_min_, *T*_max_	0.88, 0.98
No. of measured, independent and observed [*I* > 2σ(*I*)] reflections	30517, 4288, 3151
*R*_int_	0.031
(sin θ/λ)_max_ (Å^−1^)	0.676

Refinement	
*R*[*F* ^2^ > 2σ(*F* ^2^)], *wR*(*F* ^2^), *S*	0.048, 0.160, 1.11
No. of reflections	4288
No. of parameters	217
No. of restraints	26
H-atom treatment	H-atom parameters constrained
Δρ_max_, Δρ_min_ (e Å^−3^)	0.28, −0.19

Computer programs: *APEX3* and *SAINT* (Bruker, 2016 ▸), *SHELXT* (Sheldrick, 2015*a* ▸), *SHELXL2018/1* (Sheldrick, 2015*b* ▸), *DIAMOND* (Brandenburg & Putz, 2012 ▸) and *SHELXTL* (Sheldrick, 2008 ▸).

## full crystallographic data

### Crystal data

**Table:**

C_17_H_20_N_2_O_4_	*F*(000) = 1344
*M**_r_* = 316.35	*D*_x_ = 1.266 Mg m^−^^3^
Monoclinic, *C*2/*c*	Mo *K*α radiation, λ = 0.71073 Å
*a* = 23.0488 (9) Å	Cell parameters from 9996 reflections
*b* = 8.1149 (3) Å	θ = 2.3–27.3°
*c* = 18.3223 (7) Å	µ = 0.09 mm^−^^1^
β = 104.454 (1)°	*T* = 298 K
*V* = 3318.5 (2) Å^3^	Block, colourless
*Z* = 8	0.30 × 0.27 × 0.26 mm

### Data collection

**Table:**

Bruker SMART APEX CCD diffractometer	4288 independent reflections
Radiation source: fine-focus sealed tube	3151 reflections with *I* > 2σ(*I*)
Graphite monochromator	*R*_int_ = 0.031
Detector resolution: 8.3333 pixels mm^-1^	θ_max_ = 28.7°, θ_min_ = 1.8°
φ and ω scans	*h* = −31→30
Absorption correction: multi-scan (*SADABS*; Krause *et al.*, 2015)	*k* = −10→10
*T*_min_ = 0.88, *T*_max_ = 0.98	*l* = −24→24
30517 measured reflections	

### Refinement

**Table:**

Refinement on *F*^2^	Primary atom site location: structure-invariant direct methods
Least-squares matrix: full	Secondary atom site location: difference Fourier map
*R*[*F*^2^ > 2σ(*F*^2^)] = 0.048	Hydrogen site location: inferred from neighbouring sites
*wR*(*F*^2^) = 0.160	H-atom parameters constrained
*S* = 1.11	*w* = 1/[σ^2^(*F*_o_^2^) + (0.0899*P*)^2^ + 0.4855*P*] where *P* = (*F*_o_^2^ + 2*F*_c_^2^)/3
4288 reflections	(Δ/σ)_max_ = 0.001
217 parameters	Δρ_max_ = 0.28 e Å^−^^3^
26 restraints	Δρ_min_ = −0.19 e Å^−^^3^

### Special details

**Table:**

Experimental. The diffraction data were obtained from 3 sets of 400 frames, each of width 0.5° in ω, collected at φ = 0.00, 90.00 and 180.00° and 2 sets of 800 frames, each of width 0.45° in φ, collected at ω = –30.00 and 210.00°. The scan time was 20 sec/frame.
Geometry. All esds (except the esd in the dihedral angle between two l.s. planes) are estimated using the full covariance matrix. The cell esds are taken into account individually in the estimation of esds in distances, angles and torsion angles; correlations between esds in cell parameters are only used when they are defined by crystal symmetry. An approximate (isotropic) treatment of cell esds is used for estimating esds involving l.s. planes.
Refinement. Refinement of F^2^ against ALL reflections. The weighted R-factor wR and goodness of fit S are based on F^2^, conventional R-factors R are based on F, with F set to zero for negative F^2^. The threshold expression of F^2^ > 2sigma(F^2^) is used only for calculating R-factors(gt) etc. and is not relevant to the choice of reflections for refinement. R-factors based on F^2^ are statistically about twice as large as those based on F, and R- factors based on ALL data will be even larger. H-atoms attached to carbon were placed in calculated positions (C—H = 0.95 - 0.99 Å). All were included as riding contributions with isotropic displacement parameters 1.2 - 1.5 times those of the attached atoms. The ethyl group in the ester is disordered over several closely spaced sites that could not be separated so a 2-site model with ISOR restraints on the two carbon atoms was used to approximate the disorder. The geometries of the two components were restrained to be similar.

### Fractional atomic coordinates and isotropic or equivalent isotropic displacement parameters (Å^2^)

**Table:**

	*x*	*y*	*z*	*U*_iso_*/*U*_eq_	Occ. (<1)
O1	0.41842 (6)	0.43017 (16)	0.23630 (6)	0.0793 (4)	
O2	0.22537 (6)	1.02992 (16)	−0.00750 (6)	0.0805 (4)	
O3	0.41772 (5)	0.17898 (16)	0.38013 (10)	0.0912 (5)	
O4	0.51197 (4)	0.12696 (12)	0.37673 (8)	0.0698 (3)	
N1	0.42422 (5)	0.60597 (13)	0.41467 (6)	0.0471 (3)	
N2	0.43660 (5)	0.51627 (13)	0.35755 (6)	0.0486 (3)	
C1	0.40601 (6)	0.52488 (17)	0.28277 (8)	0.0535 (3)	
C2	0.36172 (6)	0.65291 (17)	0.26634 (7)	0.0509 (3)	
H2	0.340914	0.670615	0.216534	0.061*	
C3	0.34933 (5)	0.74800 (14)	0.32046 (6)	0.0426 (3)	
C4	0.38196 (5)	0.71634 (15)	0.39722 (6)	0.0438 (3)	
C5	0.30371 (6)	0.88585 (16)	0.30417 (7)	0.0517 (3)	
H5A	0.270037	0.855686	0.324288	0.062*	
H5B	0.321688	0.984256	0.330396	0.062*	
C6	0.28079 (6)	0.92540 (15)	0.22187 (7)	0.0455 (3)	
C7	0.23054 (6)	0.84826 (17)	0.17780 (8)	0.0539 (3)	
H7	0.209482	0.774610	0.200416	0.065*	
C8	0.21068 (6)	0.87733 (18)	0.10115 (8)	0.0574 (3)	
H8	0.177154	0.822651	0.072685	0.069*	
C9	0.24120 (7)	0.98837 (17)	0.06746 (8)	0.0544 (3)	
C10	0.29086 (7)	1.06967 (19)	0.11082 (8)	0.0570 (3)	
H10	0.310934	1.146522	0.088495	0.068*	
C11	0.31060 (6)	1.03723 (17)	0.18676 (8)	0.0502 (3)	
H11	0.344438	1.091021	0.214985	0.060*	
C12	0.17493 (11)	0.9506 (3)	−0.05390 (11)	0.0984 (7)	
H12A	0.166108	0.998274	−0.103401	0.148*	
H12B	0.141106	0.964302	−0.032724	0.148*	
H12C	0.183288	0.835300	−0.057109	0.148*	
C13	0.36960 (7)	0.81397 (19)	0.46090 (7)	0.0582 (4)	
H13A	0.327840	0.805916	0.459828	0.087*	
H13B	0.379875	0.927322	0.455881	0.087*	
H13C	0.393143	0.771336	0.507866	0.087*	
C14	0.48657 (6)	0.40227 (17)	0.37869 (8)	0.0528 (3)	
H14A	0.510307	0.429970	0.428760	0.063*	
H14B	0.511925	0.414544	0.343992	0.063*	
C15	0.46657 (6)	0.22591 (17)	0.37810 (9)	0.0558 (3)	
C16	0.50265 (17)	−0.0502 (2)	0.3871 (3)	0.0734 (9)	0.715 (10)
H16A	0.494832	−0.068922	0.436038	0.088*	0.715 (10)
H16B	0.468250	−0.088510	0.348723	0.088*	0.715 (10)
C17	0.55487 (19)	−0.1393 (5)	0.3817 (4)	0.1014 (15)	0.715 (10)
H17A	0.548809	−0.254816	0.388470	0.152*	0.715 (10)
H17B	0.562186	−0.121305	0.333017	0.152*	0.715 (10)
H17C	0.588697	−0.101770	0.420100	0.152*	0.715 (10)
C16A	0.4944 (4)	−0.0438 (6)	0.3548 (7)	0.0734 (9)	0.285 (10)
H16C	0.480714	−0.097642	0.394661	0.088*	0.285 (10)
H16D	0.461730	−0.043996	0.309608	0.088*	0.285 (10)
C17A	0.5442 (5)	−0.1313 (15)	0.3410 (9)	0.1014 (15)	0.285 (10)
H17D	0.532487	−0.242603	0.326694	0.152*	0.285 (10)
H17E	0.557365	−0.078443	0.301098	0.152*	0.285 (10)
H17F	0.576306	−0.131964	0.385953	0.152*	0.285 (10)

### Atomic displacement parameters (Å^2^)

**Table:**

	*U*^11^	*U*^22^	*U*^33^	*U*^12^	*U*^13^	*U*^23^
O1	0.0871 (8)	0.0886 (8)	0.0579 (6)	0.0359 (6)	0.0098 (6)	−0.0166 (6)
O2	0.1027 (9)	0.0890 (8)	0.0432 (6)	0.0049 (7)	0.0060 (6)	0.0026 (5)
O3	0.0543 (6)	0.0670 (7)	0.1584 (14)	0.0010 (5)	0.0378 (7)	0.0124 (8)
O4	0.0504 (6)	0.0479 (5)	0.1109 (9)	0.0018 (4)	0.0199 (6)	−0.0016 (5)
N1	0.0496 (6)	0.0486 (6)	0.0416 (5)	−0.0019 (4)	0.0085 (4)	0.0021 (4)
N2	0.0494 (6)	0.0477 (6)	0.0465 (6)	0.0068 (4)	0.0079 (5)	0.0020 (4)
C1	0.0559 (7)	0.0569 (7)	0.0467 (7)	0.0103 (6)	0.0107 (6)	−0.0031 (5)
C2	0.0572 (7)	0.0545 (7)	0.0382 (6)	0.0099 (6)	0.0067 (5)	0.0000 (5)
C3	0.0457 (6)	0.0419 (6)	0.0403 (6)	0.0004 (5)	0.0109 (5)	0.0030 (4)
C4	0.0481 (6)	0.0452 (6)	0.0382 (6)	−0.0033 (5)	0.0113 (5)	0.0016 (4)
C5	0.0603 (8)	0.0492 (7)	0.0466 (7)	0.0110 (6)	0.0150 (6)	0.0022 (5)
C6	0.0479 (6)	0.0420 (6)	0.0468 (6)	0.0087 (5)	0.0125 (5)	0.0032 (5)
C7	0.0512 (7)	0.0477 (7)	0.0618 (8)	−0.0010 (5)	0.0120 (6)	0.0075 (6)
C8	0.0497 (7)	0.0550 (8)	0.0604 (8)	0.0004 (6)	0.0002 (6)	−0.0032 (6)
C9	0.0619 (8)	0.0554 (7)	0.0445 (7)	0.0104 (6)	0.0103 (6)	0.0008 (5)
C10	0.0625 (8)	0.0588 (8)	0.0531 (8)	−0.0039 (6)	0.0210 (6)	0.0047 (6)
C11	0.0460 (6)	0.0532 (7)	0.0514 (7)	−0.0034 (5)	0.0120 (5)	−0.0023 (5)
C12	0.1217 (17)	0.0963 (14)	0.0571 (10)	0.0208 (12)	−0.0154 (10)	−0.0184 (9)
C13	0.0673 (9)	0.0660 (8)	0.0411 (7)	0.0043 (7)	0.0130 (6)	−0.0044 (6)
C14	0.0451 (7)	0.0519 (7)	0.0578 (8)	0.0046 (5)	0.0064 (6)	0.0050 (6)
C15	0.0469 (7)	0.0528 (7)	0.0662 (9)	0.0044 (6)	0.0112 (6)	0.0040 (6)
C16	0.0679 (13)	0.0496 (9)	0.100 (3)	−0.0014 (8)	0.0153 (17)	0.0002 (11)
C17	0.0807 (19)	0.0606 (12)	0.159 (4)	0.0052 (12)	0.022 (3)	−0.018 (3)
C16A	0.0679 (13)	0.0496 (9)	0.100 (3)	−0.0014 (8)	0.0153 (17)	0.0002 (11)
C17A	0.0807 (19)	0.0606 (12)	0.159 (4)	0.0052 (12)	0.022 (3)	−0.018 (3)

### Geometric parameters (Å, º)

**Table:**

O1—C1	1.2325 (16)	C9—C10	1.386 (2)
O2—C9	1.3722 (17)	C10—C11	1.3770 (19)
O2—C12	1.413 (3)	C10—H10	0.9300
O3—C15	1.1980 (17)	C11—H11	0.9300
O4—C15	1.3243 (17)	C12—H12A	0.9600
O4—C16A	1.471 (3)	C12—H12B	0.9600
O4—C16	1.473 (2)	C12—H12C	0.9600
N1—C4	1.3030 (16)	C13—H13A	0.9600
N1—N2	1.3623 (15)	C13—H13B	0.9600
N2—C1	1.3773 (17)	C13—H13C	0.9600
N2—C14	1.4525 (16)	C14—C15	1.503 (2)
C1—C2	1.4347 (18)	C14—H14A	0.9700
C2—C3	1.3423 (17)	C14—H14B	0.9700
C2—H2	0.9300	C16—C17	1.428 (3)
C3—C4	1.4428 (16)	C16—H16A	0.9700
C3—C5	1.5131 (17)	C16—H16B	0.9700
C4—C13	1.4953 (17)	C17—H17A	0.9600
C5—C6	1.5027 (18)	C17—H17B	0.9600
C5—H5A	0.9700	C17—H17C	0.9600
C5—H5B	0.9700	C16A—C17A	1.424 (4)
C6—C7	1.3850 (19)	C16A—H16C	0.9700
C6—C11	1.3894 (18)	C16A—H16D	0.9700
C7—C8	1.384 (2)	C17A—H17D	0.9600
C7—H7	0.9300	C17A—H17E	0.9600
C8—C9	1.380 (2)	C17A—H17F	0.9600
C8—H8	0.9300		
			
C9—O2—C12	117.63 (16)	O2—C12—H12B	109.5
C15—O4—C16A	114.3 (4)	H12A—C12—H12B	109.5
C15—O4—C16	116.62 (16)	O2—C12—H12C	109.5
C4—N1—N2	117.76 (10)	H12A—C12—H12C	109.5
N1—N2—C1	125.72 (10)	H12B—C12—H12C	109.5
N1—N2—C14	116.08 (10)	C4—C13—H13A	109.5
C1—N2—C14	118.20 (11)	C4—C13—H13B	109.5
O1—C1—N2	120.40 (12)	H13A—C13—H13B	109.5
O1—C1—C2	125.62 (13)	C4—C13—H13C	109.5
N2—C1—C2	113.97 (11)	H13A—C13—H13C	109.5
C3—C2—C1	122.19 (12)	H13B—C13—H13C	109.5
C3—C2—H2	118.9	N2—C14—C15	112.53 (11)
C1—C2—H2	118.9	N2—C14—H14A	109.1
C2—C3—C4	117.55 (11)	C15—C14—H14A	109.1
C2—C3—C5	123.01 (11)	N2—C14—H14B	109.1
C4—C3—C5	119.44 (11)	C15—C14—H14B	109.1
N1—C4—C3	122.52 (11)	H14A—C14—H14B	107.8
N1—C4—C13	116.68 (11)	O3—C15—O4	124.13 (14)
C3—C4—C13	120.78 (11)	O3—C15—C14	126.27 (13)
C6—C5—C3	114.13 (10)	O4—C15—C14	109.58 (11)
C6—C5—H5A	108.7	C17—C16—O4	109.4 (3)
C3—C5—H5A	108.7	C17—C16—H16A	109.8
C6—C5—H5B	108.7	O4—C16—H16A	109.8
C3—C5—H5B	108.7	C17—C16—H16B	109.8
H5A—C5—H5B	107.6	O4—C16—H16B	109.8
C7—C6—C11	117.61 (12)	H16A—C16—H16B	108.2
C7—C6—C5	121.46 (12)	C16—C17—H17A	109.5
C11—C6—C5	120.90 (12)	C16—C17—H17B	109.5
C8—C7—C6	122.08 (13)	H17A—C17—H17B	109.5
C8—C7—H7	119.0	C16—C17—H17C	109.5
C6—C7—H7	119.0	H17A—C17—H17C	109.5
C9—C8—C7	119.25 (13)	H17B—C17—H17C	109.5
C9—C8—H8	120.4	C17A—C16A—O4	109.9 (7)
C7—C8—H8	120.4	C17A—C16A—H16C	109.7
O2—C9—C8	124.76 (14)	O4—C16A—H16C	109.7
O2—C9—C10	115.59 (14)	C17A—C16A—H16D	109.7
C8—C9—C10	119.63 (13)	O4—C16A—H16D	109.7
C11—C10—C9	120.36 (13)	H16C—C16A—H16D	108.2
C11—C10—H10	119.8	C16A—C17A—H17D	109.5
C9—C10—H10	119.8	C16A—C17A—H17E	109.5
C10—C11—C6	121.05 (13)	H17D—C17A—H17E	109.5
C10—C11—H11	119.5	C16A—C17A—H17F	109.5
C6—C11—H11	119.5	H17D—C17A—H17F	109.5
O2—C12—H12A	109.5	H17E—C17A—H17F	109.5
			
C4—N1—N2—C1	−3.99 (18)	C5—C6—C7—C8	−176.68 (12)
C4—N1—N2—C14	176.09 (11)	C6—C7—C8—C9	−1.0 (2)
N1—N2—C1—O1	−175.15 (13)	C12—O2—C9—C8	−1.5 (2)
C14—N2—C1—O1	4.8 (2)	C12—O2—C9—C10	−179.82 (15)
N1—N2—C1—C2	6.4 (2)	C7—C8—C9—O2	−178.74 (13)
C14—N2—C1—C2	−173.70 (12)	C7—C8—C9—C10	−0.4 (2)
O1—C1—C2—C3	177.80 (15)	O2—C9—C10—C11	180.00 (13)
N2—C1—C2—C3	−3.8 (2)	C8—C9—C10—C11	1.5 (2)
C1—C2—C3—C4	−0.6 (2)	C9—C10—C11—C6	−1.3 (2)
C1—C2—C3—C5	178.73 (13)	C7—C6—C11—C10	−0.12 (19)
N2—N1—C4—C3	−1.25 (17)	C5—C6—C11—C10	177.83 (12)
N2—N1—C4—C13	−179.54 (11)	N1—N2—C14—C15	105.60 (13)
C2—C3—C4—N1	3.40 (18)	C1—N2—C14—C15	−74.33 (16)
C5—C3—C4—N1	−175.96 (11)	C16A—O4—C15—O3	17.8 (6)
C2—C3—C4—C13	−178.39 (12)	C16—O4—C15—O3	−7.2 (3)
C5—C3—C4—C13	2.25 (18)	C16A—O4—C15—C14	−163.6 (6)
C2—C3—C5—C6	−9.45 (19)	C16—O4—C15—C14	171.4 (3)
C4—C3—C5—C6	169.88 (11)	N2—C14—C15—O3	−18.8 (2)
C3—C5—C6—C7	90.46 (16)	N2—C14—C15—O4	162.61 (12)
C3—C5—C6—C11	−87.41 (15)	C15—O4—C16—C17	178.2 (2)
C11—C6—C7—C8	1.3 (2)	C15—O4—C16A—C17A	168.9 (7)

### Hydrogen-bond geometry (Å, º)

*Cg*1 is the centroid of the C1–C4/N1/N2 ring.

**Table:**

*D*—H···*A*	*D*—H	H···*A*	*D*···*A*	*D*—H···*A*
C14—H14*B*···O1^i^	0.97	2.44	3.4041 (19)	175
C16—H16*B*···*Cg*1^ii^	0.97	2.86	3.586 (3)	132

Symmetry codes: (i) −*x*+1, *y*, −*z*+1/2; (ii) *x*, *y*−1, *z*.
